# Crowdfunding and global health disparities: an exploratory conceptual and empirical analysis

**DOI:** 10.1186/s12992-019-0519-1

**Published:** 2019-11-28

**Authors:** Nora J. Kenworthy

**Affiliations:** School of Nursing and Health Studies, University of Washington, Bothell, USA

**Keywords:** Crowdfunding, Medical crowdfunding, Global health crowdfunding, Political determinants of health, Technology and health

## Abstract

**Background:**

The use of crowdfunding platforms to cover the costs of healthcare is growing rapidly within low-, middle-, and high-income countries as a new funding modality in global health. The popularity of such “medical crowdfunding” is fueled by health disparities and gaps in health coverage and social safety-net systems. Crowdfunding in its current manifestations can be seen as an antithesis to universal health coverage. But research on medical crowdfunding, particularly in global health contexts, has been sparse, and accessing robust data is difficult. To map and document how medical crowdfunding is shaped by, and shapes, health disparities, this article offers an exploratory conceptual and empirical analysis of medical crowdfunding platforms and practices around the world. Data are drawn from a mixed-methods analysis of medical crowdfunding campaigns, as well as an ongoing ethnographic study of crowdfunding platforms and the people who use them.

**Results:**

Drawing on empirical data and case examples, this article describes three main ways that crowdfunding is impacting health equity and health politics around the world: 1) as a technological determinant of health, wherein data ownership, algorithms and platform politics influence health inequities; 2) as a commercial determinant of health, wherein corporate influence reshapes healthcare markets and health data; 3) and as a determinant of health politics, affecting how citizens view health rights and the future of health coverage.

**Conclusions:**

Rather than viewing crowdfunding as a social media fad or a purely beneficial technology, researchers and publics must recognize it as a complex innovation that is reshaping health systems, influencing health disparities, and shifting political norms, even as it introduces new ways of connecting and caring for those in the midst of health crises. More analysis, and better access to data, is needed to inform policy and address crowdfunding as a source of health disparities.

## Introduction

In September 2018, a frenzy broke out on Twitter as Zimbabwe’s recently-appointed Finance Minister Mthuli Ncube put out an emergency message to citizens regarding the country’s cholera outbreak. Rather than informing citizens about preventative measures or treatment centers, Ncube’s tweet asked them to contribute funds to the national response: “Together with my colleagues at Min of Health, we have set up an auditable emergency crowdfund to further efforts to fight cholera to date,” he wrote [1]. Exhausted by years of corruption, financial mismanagement, and poor public services, Zimbabweans quickly lashed back [2]. Given that Harare’s failure to provide adequate water supplies to residents was suspected as the cause of the outbreak [3], the crowdfunding appeal appeared to many as an “irresponsible, insensitive and indefensible act” [2].

Three months later across the Atlantic, far-right supporters of US President Donald Trump began several crowdfunding campaigns to fund a fortified wall between the USA and Mexico, aimed at deterring and preventing migrants—many of them intending to claim asylum—from reaching US territory. Citizens turned to crowdfunding while President Trump shut down the government over the absence of legislative support for border wall funding. In less than a month, the most prominent campaign started by Brian Kolfage had raised more than $18 million. While far short of the $1 billion goal Kolfage had set, or the $5 billion President Trump was demanding from Congress [4], it quickly became the second-highest earning campaign in the history of the popular crowdfunding site GoFundMe.^1^ As the government shutdown dragged on, federal workers also turned to crowdfunding to cover their lost wages; donors to these campaigns said that it was “something constructive” to do “when [people] feel powerless” [5].

Despite abundant differences, these two campaigns embody the political anxieties, aspirations, and griefs of citizens. They underscore the ubiquity of crowdfunding, as it has seeped into public life across the globe, often serving as a replacement for, or augment to, government programs. More commonly, however, crowdfunding involves a personal appeal in response to individual crisis: the majority of campaigns in the US are for medical or health-related fundraising [6, 7]. The rise of medical crowdfunding is fueled by gaps in the social safety net, rising healthcare costs and debts, and inadequate access to healthcare coverage [8, 9]. Whether in Zimbabwe or in, say, South Carolina, the normalization of medical crowdfunding as a means of accessing healthcare or avoiding financial ruin reflects the global entrenchment of neoliberalism and fiscal austerity, and the individualized responsibilization and self-marketing which these political economies foster. The Zimbabwe cholera and US border wall campaigns also underscore how crowdfunding is becoming a tool of political projects, a means of political expression, and a political determinant of health. Such campaigns emerge in political-economic worlds where influence is counted in dollars and where precarity, and even state abandonment, has become normalized. As populations across the globe increasingly turn to online platforms that harness the small charities of the crowd to patch over enormous gaps in the social system, it is essential to examine how these platforms are creating new political realities and reshaping norms regarding who deserves assistance, under what terms, and how it will be provided.

There is a particular need for research on crowdfunding as a global phenomenon with broader effects on health equities and politics worldwide [10]. Little is known about how crowdfunding may exacerbate health disparities by influencing who can and cannot afford care. As with other technologies, it is important to recognize crowdfunding as a likely social determinant of health. This article identifies three powerful domains in which crowdfunding may impact healthcare inequities: 1) as a technological determinant of health, where algorithms, platform politics and data ownership policies influence healthcare inequities; 2) as a commercial determinant of health, where corporate practices and influence reshape healthcare markets, data use, and profiteering; and 3) as a determinant of health politics, shaping how citizens view health rights and the future of health coverage. Following work by the Lancet–University of Oslo Commission on Global Governance for Health, which identified global power imbalances, neoliberalism, inadequate regulation of transnational corporations, and marketization of health systems as key political determinants of health around the world [11], this article offers an exploratory conceptual and empirical analysis of crowdfunding as a determinant of health which reflects many of these concerns.

Critics of the Oslo Commission have argued that its recommendations did not fully acknowledge how power, political processes, corporate influence, and neoliberal ideologies shape health outcomes [12]. As scholars continue to examine these aspects of the political determinants of health, global governance institutions and researchers have missed opportunities to identify and tackle root political causes [13, 14]. This article aims to remain explicitly attuned to power, neoliberalism, and corporate influence while examining the impacts of crowdfunding on healthcare by analyzing the history and current trends of the medical crowdfunding industry worldwide. To ensure that this research is relevant to global governance efforts, it employs an anticipatory framework for explaining a rapidly emerging industry, recognizing that crowdfunding research must investigate not only past and current trends, but future possibilities and as-yet-undocumented potential sources of health inequities. In pursuing such an anticipatory frame of analysis, the article draws on studies of related social media and technology platforms that have documented similar social and health impacts. Finally, the conclusion identifies opportunities for regulating industry and increasing public access to data, to ensure more robust research in the future.

## Background

### The crowdfunding industry

Crowdfunding involves a financial appeal to social networks, often using internet-based platforms and social media to amplify the spread of the appeal [15]. Although crowdfunding first gained attention as a means for “consumer-investors” to support inventions and art projects through platforms like Kickstarter, donation-based crowdfunding has grown prodigiously in recent years in many regions of the world [16–19]. In contrast with equity and reward crowdfunding, donation-based crowdfunding is used by campaigners to appeal for financial help without formal expectations of reciprocity. Personal medical and health appeals have consistently been the leading type of campaign on popular donation-based crowdfunding websites like GoFundMe [6, 7]. By 2018, GoFundMe reported that it hosted more than 250,000 medical campaigns per year across 19 countries, which in total raised more than $650 million, and comprised one out of every three campaigns on the site [20, 21].

While GoFundMe dominates the donation-based crowdfunding marketplace in its 19 supported countries, all of which are in North America and Western Europe, crowdfunding has become a remarkably global phenomenon [22, 23]. Similarly popular platforms target lower- and middle-income countries, as with the platforms Ketto in India (co-founded by Bollywood star Kunal Kapoor), BackaBuddy in South Africa, and M-Changa in Kenya [18, 19, 24, 25]. GoFundMe, M-Changa and other platforms can be described as **peer-based crowdfunding** in that they largely aim to connect individuals seeking donations with others in their immediate or extended networks who can donate to personal causes.^2^ On these platforms, campaigns are typically started by an individual in need or persons within his or her close network. Most platforms in this category are for-profit entities, raising money from fees or “tips” on donations given to campaigns.

In the field of global health, a second type of donation-based crowdfunding is increasingly popular: it aims to connect individual donors in mostly high-income countries with patients or projects in need of funding in low-or middle-income countries. Such **philanthropic crowdfunding** is hosted by platforms such as Watsi and Caringcrowd, and is often explicitly focused on global health causes. Watsi, for example, features profiles of patients in need of critical healthcare services at partner medical institutions throughout the Global South, enabling donors (typically located in the Global North) to select the patient recipients they would like to fund [26]. Caringcrowd takes a different approach, featuring public health projects from around the world that are carefully curated by a panel of experts. What these philanthropic crowdfunding platforms have in common is the relative geopolitical and economic distance between donors and recipients: in contrast to peer-based crowdfunding, campaigns are initiated by (often distant) medical, nonprofit, and expert entities, rather than patients and their close social networks.

### Crowdfunding and health inequities

Across this diverse and rapidly growing global marketplace, several key dynamics remain constant. Medical crowdfunding is fueled by gaps in healthcare coverage and access, underscoring how the lack of universal health coverage (UHC) creates additional, unexpected health disparities [8, 22, 27]. In the USA, our previous research documents a disproportionately large number of medical campaigns in states that had not accepted the expansion of public benefits such as Medicaid under the Affordable Care Act (ACA) [8]. And as Snyder has pointed out, by appearing to successfully address individual needs, crowdfunding campaigns can exacerbate systemic injustice by “[reducing] pressure for systemic reforms” [28]. However, it is worth underscoring that crowdfunding is a poor substitute for more robust forms of health coverage. Our research found that only about 10% of campaigns meet their financial goals—goals that frequently *understate* actual financial needs [8].

While inequities in access and coverage drive people to crowdfunding platforms, the logics, norms, and processes embedded within platforms may serve to exacerbate inequities. Crowdfunding is a distinctly “downstream” technology in that it works best to address acute problems with a clear, often biomedical, solution. Watsi, for example, funds only patients with treatable conditions who need a one-time medical intervention with ‘a high probability of success’ costing less than $1500, such as simple orthopedic surgery [26]. On GoFundMe we have found that campaigns with discrete, solvable problems appear to be more successful and fit crowdfunding platform logics better; as a result, persons with more chronic or complicated conditions struggle to articulate stories and gain attention, in hopes of getting the help they need [8]. Individuals with complex, overlapping needs often appear less deserving or as having needs beyond the scope of a standalone campaign [8]. Thus, the more generalized one’s distress or the more complex one’s needs, the more difficult it is to achieve crowdfunding success. This means that the human crises brought about by structural violence—complex, multiplicative, chronic, overlapping—are least likely to be adequately ameliorated by crowdfunding. By targeting solvable problems among a select, 'deserving' few, most crowdfunding not only fails to fill gaps in health coverage: it also conceals and exacerbates the structural violence of austerity and inadequate social safety nets that fuel health disparities.

Research on medical crowdfunding has highlighted the potential of this new media form to create unfairnesses and pose ethical challenges for users, their social networks, and society more broadly [8, 28–32]. However, very little research has documented how health disparities shape access to, and outcomes from, crowdfunding campaigns—indeed, how crowdfunding itself may fuel health disparities [27, 33]. One notable exception, a 2019 study of Canadian cancer campaigns, found use of crowdfunding to be densest in wealthy urban areas with high education levels [34]. At its core, crowdfunding provides a technological infrastructure that amplifies individual choices in determining who gets financial support for health needs. The privileging of individual choice introduces ample room for biases, judgements, and discrimination; and, as described below, these dynamics can be exacerbated by platform dynamics, algorithms, and data ownership practices. Any crowdfunding campaign can be subjected to myriad judgements from potential donors about perceived deservingness and the “value” of donations for assistance. Campaigners may face judgement or discrimination for fundraising for health conditions that are often publicly perceived as being the result of risky, dangerous, or irresponsible behaviors, including conditions linked to obesity, smoking, or sexual activity.

Campaigns are also read through the lenses of social biases: platforms take on, and at times amplify, the racial, economic, and cultural injustices of their social contexts. Take, for example, the challenges of developing a crowdfunding campaign as a poor black woman in the USA, where decades of social policies, political rhetoric, and media portrayals have reinforced the idea that black women, especially those living in poverty, are undeserving of social assistance [35–37]. Systematic investigations of how these dynamics impact crowdfunding access and outcomes have been sparse, and this article will discuss some of the technological and political barriers to more robust research on the topic. Further, although recent research has revealed how platforms such as Facebook, YouTube, and Google have struggled to understand how complex social and cultural dynamics combine with algorithms and platform dynamics to promote hateful, violent, and politically damaging content, more research is needed on how similar dynamics may operate invisibly on crowdfunding sites in ways that exacerbate health disparities [38–40]. This article looks upstream at how platforms are designed, managed, marketed, and governed, in order to explain crowdfunding as a technological, commercial, and political determinant of health.

## Methods

This article synthesizes research findings from several interlinked research projects examining medical crowdfunding in different settings. The first is a long-term in-person and online ethnography of US medical crowdfunding, which includes interviews with industry leaders, patients, and crowdfunders, paired with an observations and analysis of related sites, discourses, and media. The second project is a study of global health crowdfunding, including an ongoing analysis of global crowdfunding platforms and an in-depth case study on Watsi. The third project involves mixed-methods analysis of several randomized samples of US medical crowdfunding campaigns between 2011 and 2016, with quantitative analysis of data on campaign success and spread, as well as a qualitative analysis of text, photos, and videos in 200 campaigns to assess variables such as deservingness, identity, illness experience, debt, and health insurance experiences. Readers are referred to other published works on these projects for more in-depth descriptions of methods [8, 26].

For the purposes of this article, lessons and data are drawn from the above projects and combined with results from a new survey of popular global health and medical crowdfunding sites. This survey compiled a list of popular, donation-based crowdfunding platforms from published studies, online searches, and market analyses [18, 22, 24, 41]. Crowdfunding platforms that were not donation-based or did not host medical or health-related campaigns were excluded. Each platform was then analyzed for the types of crowdfunding platform used; countries served; types of causes hosted on the platform; the status of the platform as a for-profit or non-profit venture; any known ties to industry, corporations, or for-profit ventures; and, given the considerable fluctuation in the marketplace, each platform’s status as of January 2019. Data for this survey were compiled from publicly-available sources: the platforms and websites themselves, any publicly available documents (annual reports, press releases, company profiles); news reports, internet archives of previous website pages accessed through the Wayback Machine, and available documentation on their registration as corporations or non-profit entities in relevant countries. The findings of this survey are provided in Additional file 1.

## Results and discussion

### Crowdfunding as a technological determinant of health

Technology has generally been perceived as a social good within the field of healthcare, with policymakers, administrators and even researchers lauding its ability to bridge, rather than widen, health disparities [42, 43]. However, scholars are increasingly warning that existing social disparities—especially regarding media literacies, social capital, education levels and access to technology infrastructures—can deeply impact patients’ capacities to use and gain benefit from health technologies [44, 45]. Public recognition is also growing about ways in which technologies may serve to exacerbate inequities, highlighting how technology can and should be recognized as a determinant of health. Central questions concern who creates and owns data, as well as data protection, access, and further sale —and how existing social inequities shape these practices [46, 47]. Researchers have also highlighted the role of algorithms and artificial intelligence—often created by developers in an industry with longstanding issues with diversity and inclusion—in exacerbating or amplifying racism, inequities, and biases [38–40]. Taken together, these concerns underscore the ways that online and social media landscapes are neither wholly egalitarian public spaces, nor marked by a clear digital divide between those who can and cannot access them. What is becoming clear is that these are highly uneven media landscapes that consumers must traverse in order to participate in the information economy; that the harmful effects of these landscapes accrue disproportionately among those who are most vulnerable or most likely to be discriminated against; and that few consumers have the expert knowledge or tools necessary to ensure their own safety or protect themselves against harm.

There is an urgent need, particularly in global health, to recognize technology as a determinant of health. Medical crowdfunding is one such technological determinant: it operates as a social media platform that mediates patients’ access to key health goods, depending on their success in fundraising. The industry’s platform design, use of algorithms, and integration with other forms of social and traditional media result in a highly competitive marketplace that exacerbates inequities by creating winners and losers. This section explores these factors and their impacts on health inequity; the following section describes how commercialization, marketization, and data privatization further exacerbate these inequities.

Part of the magic of online platforms, as media scholars often remind us, is that they can appear to be the same for everyone—an egalitarian public space—while creating entirely different realities depending on one’s social location, one’s various forms of capital, and the biases one faces [39, 40]. Most patients who set up a crowdfunding campaign are provided with the same page format and opportunities to post and share information. However, the information that can be shared, the very appearance and appeal of the page, will depend on a wide set of determinants often beyond their control—ranging from the kind of computer or mobile device they use to set up the page, to the media literacy skills they draw on to craft a story and post pictures and video. Disabilities also pose particular kinds of challenges for using social media and websites, and many platforms are poorly designed to accommodate persons with disabilities. There are also many medical conditions (such as comas and strokes) which make it difficult, if not impossible, to use crowdfunding sites when one is actually experiencing a medical crisis. In fact, those who have been recently or temporarily disabled by a health condition may face the most barriers in accessing crowdfunding technology without outside help, because they are in the process of adjusting to new disabilities and may not be aware of assistive technology tools and devices.

Even those who are successful in crowdfunding can find that it creates distinct vulnerabilities for them—particularly if they rely on public assistance, have stigmatized conditions, or are likely to face discrimination as their campaign gains visibility. In places like the USA, where public benefits are tied to one’s level of income and financial assets, crowdfunders can lose access to their benefits because funds raised can put them over the qualifying income levels for social services [48]. Crowdfunding for stigmatized illnesses like HIV/AIDS can involve socially dangerous or deeply uncomfortable public disclosures of status. A randomized sample of 200 US crowdfunding campaigns included several for people who were publicly disclosing their HIV status, infertility, and struggles with addiction for the first time in order to crowdfund. Because campaigns are public and can be shared by anyone, campaigners have little control over who sees these disclosures, and may face unanticipated invasions of privacy, discrimination, or unintended consequences like job loss [49]. Disclosures from campaigns also persist online long after campaigners have created them—there are multiple steps users must take to remove content from platforms. Moreover, even campaigns removed from sites can remain in internet archives, or in archived data scraped by institutions or individuals. Campaigns that gain considerable visibility or “go viral” can also present challenges to those who are more likely to face discrimination online. For example, a black woman who ran an unexpectedly successful campaign in Seattle described how the campaign’s success brought unwelcome invasions of her privacy and questions from strangers about her credibility—experiences that white organizers of similarly successful campaigns did not report in our interviews.

Algorithms used by crowdfunding sites to return search results and highlight trending campaigns amplify these inequities. Because algorithms are rarely disclosed to the public (see below), assessing how they specifically shape campaign visibility is difficult. Many algorithms on social media sites are designed to show content that is popular; as a result, an already trending campaign or post gains more and more attention, while those that have received little attention get pushed further down in search results or feeds. Four years of close ethnographic observation of several large crowdfunding platforms revealed that main webpages, medical campaign listings, and search results of these sites prioritize “trending” and more successful campaigns, as well as those with geographic proximity to site visitors. This essentially renders all but the most successful campaigns invisible to the casual visitor: most campaigns circulate only within limited spheres of users’ social networks, probably contributing to the mere 10% of campaigns that reach their financial goals, and the select few that “go viral.” Platforms such as GoFundMe are also highly integrated with other social media sites and search engines, because users attract attention for their campaigns by spreading the word across other social media platforms, where similar algorithms shape content visibility. Research on these other platforms has highlighted how algorithms, shaped by human and institutional biases, exacerbate and fuel discrimination [40, 50, 51]. Thus, algorithmic inequities faced by crowdfunding users multiply as campaigns rely on different platforms for visibility and spread.

Successful crowdfunders are aware of these dynamics and use them to their advantage. In interviews, these crowdfunders describe planning for weeks or months before campaigns launch, and lining up large networks of friends who are ready to donate and share the campaign as soon as it goes online, in order to increase its visibility as a “trending” campaign. They set up multiple social media accounts to amplify their messages, and often rely on friends working in the media or public relations to help manage their social media messaging. Parallel industries have also arisen alongside crowdfunding with the explicit purpose of helping to popularize campaigns. Accounts on Twitter with thousands of followers can be paid to promote campaigns, and GoFundMe itself contracts with public relations firms to spread the word about trending campaigns. GoFundMe has also been highly successful at leveraging more traditional news media to direct traffic to campaigns, including developing a corporate partnership with the *San Diego Union-Tribune* to run regular news stories linked to GoFundMe campaigns [52].

Digital and technological disparities are often thought of as a matter of skills deficits. However, a more comprehensive analysis of technological determinants of health reveals that disparities are often shaped by platforms and algorithms, as well as users’ resources and access to forms of networked capital that can exploit these dynamics. Taken together, these technological determinants compound to create a unique market of “deservingness,” where certain people face nearly insurmountable barriers to success. As described in the following sections, these technological determinants are compounded by commercial and political determinants of health, including the ownership and privatization of data, and the ways in which social media visibility translates into political power.

### Crowdfunding as a commercial determinant of health

While research on the commercial determinants of health has emphasized the impacts of corporate influence through marketing, lobbying, social responsibility efforts and supply-chain management, there are broader impacts of corporate influence, such as their capture of global governance institutions and power to shift social norms and practices, that must also be recognized [53, 54]. Further, corporations have played a significant role in the broader expansion and normalization of marketized solutions to health, the promotion of philanthrocapitalism in global health, and the entrenchment of neoliberal values in healthcare policies, services, and practices. Crowdfunding is a powerful commercial determinant of health, because crowdfunding platforms wield direct influence through everyday practices while also playing a powerful role in the shifting of norms, the privatization of public assets, the marketization of health and the further institutionalization of philanthrocapitalist solutions to global health challenges. Crowdfunding has become a central pillar of a larger “global expansion of care” under what Mitchell and Sparke have called the New Washington Consensus [55]. Here, philanthrocapitalist care projects do not run counter to capitalism, but actually protect it, “cultivating new market subjects” [55]. Crowdfunding is also part of a broader incursion of technological solutions and platforms into health sectors around the world, particularly as countries pursue UHC goals. This section focuses on the industry activities most likely to influence population health disparities.

The majority (68%) of crowdfunding platforms identified by this study are run by private, for-profit entities (see Additional file 1). Crowdfunding is a highly profitable industry: platforms generate revenue from fees or “tips” on donated funds, the collection and sale of user data, advertising, and cross-platform marketing strategies. As the crowdfunding marketplace has expanded, more successful firms have aggressively acquired competitors, decreasing competition and often edging out non-profit platforms. GoFundMe recently acquired several competitor sites, including YouCaring and Crowdrise; even prior to these acquisitions, it was estimated to control 90% of the US, and 80% of the global, social crowdfunding market [56], with some $100 million in annual revenue [57]. But GoFundMe is not alone: Leetchi, a leading European for-profit crowdfunding platform with more than 12 million users, has used its crowdfunding expertise to start a web-based money transfer program. Some crowdfunding sites also raise revenues by selling platform architectures and expertise to other companies seeking to host crowdfunding on their own websites. This rapidly expanding industry has also given birth to for-profit firms such as the consulting firm AlliedCrowds, which aims at “disrupting development” by selling expensive crowdfunding architecture and development expertise to developers in emerging markets [58].

Most ostensibly non-profit platforms are organized as public–private partnerships and/or have deep ties to industry partners (see Additional file 1). Global Giving, for example, was started in the early 2000s by two World Bank colleagues, Mari Kuraishi and Dennis Whittle, and is often described as the world’s first crowdfunding platform. Though it is listed as a non-profit in both the US and the UK, it lists nearly 200 corporate partners on its website. Through these partnerships—which include the alcohol and sugar-sweetened beverage industries, pharmaceutical companies, and tech and financial firms—Global Giving is heavily embedded in a corporate social responsibility (CSR) industry that serves to obscure and distract from corporate harms and commercial determinants of poor health [59, 60]. Global Giving is not alone in relying on such partnerships. Caringcrowd, self-described as “*the* crowdfunding platform for global public health,” is wholly owned (or in its own words, “powered”) by Johnson & Johnson. Most non-profit platforms thus serve as a powerful means for corporations to engage in CSR activities that not only enable the obfuscation of corporate harms, but also reduce corporations’ tax burdens, undermining public investment in the very social programs they claim to supplement.

These dynamics blur the lines between crowdfunding’s often-stated purpose of democratizing aid and promoting “public health, powered by the people” and the industry’s upstream impacts on health [61]. Crowdfunding is poorly suited for addressing the determinants of health or providing preventative services; nor is it designed to strengthen public health infrastructures or broaden universal health coverage. As GoFundMe itself has stated, “[While we] can provide timely, critical help to people facing health care crisis, we do not aim to be a substitute social safety net” [62]. Regardless of whether GoFundMe wishes to be a safety net, its profits derive directly from the inadequacies of public safety nets, and its activities undermine them. This phenomenon is not limited to peer-based crowdfunding: Watsi specifically targets and hosts campaigns for patients in need of expensive, acute medical treatment, supporting non-profit and public–private partners to provide such services in the absence of more robust state healthcare. Caringcrowd largely funnels money into non-governmental or individually-led projects that attempt to fill gaps in public health systems and infrastructure. While important (and sometimes more ‘upstream’ in approach than other platforms), such funding mechanisms may contribute to the widely-criticized “NGO-ization” of the public sphere in the Global South [63].

Crowdfunding marketplaces directly impact health through their data ownership, use, and transparency practices, which are critical commercial determinants of health in the digital economy. For example, GoFundMe campaigners are encouraged to post personal details, including highly sensitive health data, to make their stories credible. This is then combined with data that GoFundMe collects from users, including extensive information from social media profiles and location data from IP addresses. Consumers have little way of knowing to whom their personal data are sold, or even the purposes of such sales. Because data persist long after users have written campaigns and forgotten about them, data have future values and uses that are hard to predict. And because all campaigns on GoFundMe are publicly accessible to all, in order to ensure maximum spread, users have no way of shielding their sensitive health information from anyone who might seek to use it. This enables other corporations to access data even if GoFundMe has not sold such information. These possibilities have the potential to directly impact population health and undermine UHC efforts. For example, if the US government overturns protections for patients with pre-existing conditions enacted under the Affordable Care Act, data on crowdfunding campaign pages could be used by health insurers to discover pre-existing conditions or other past choices that would justify excluding customers from health coverage. Paradoxically, in the absence of universal coverage, this introduces the possibility that crowdfunding campaigns which enable users to access needed healthcare today may become the evidence used to deny them access to care in the future.

Data, often referred to as “the new oil” in the fields of global health and development [64], holds significant value for for-profit crowdfunding companies, and are thus closely guarded against use by industry competitors as well as researchers or public agencies. While individual campaign pages are publicly accessible, GoFundMe’s Terms and Conditions specify that users may not “modify, copy, frame, scrape, rent, lease, loan, sell, distribute or create derivative works based on the Services or the Services Content, in whole or in part,” except for the content they add themselves [65]. They also expressly prohibit any use of “data mining, robots, scraping or similar data gathering or extraction methods.” Thus, as patients increasingly turn towards private crowdfunding marketplaces to fill gaping holes in social safety nets, health data are shifting from public repositories to private platforms. Consequently, ways of knowing and measuring the potential inequities produced by crowdfunding are limited by data privatization and ownership policies. Algorithms that are key technological determinants of health inequity are also heavily guarded from public or even government scrutiny. As highlighted by those who monitor trade agreements, technology companies work to ensure that extensive protections of their source code are written into new trade agreements, so that even governments cannot scrutinize their algorithms—even when investigating cases of suspected systemic discrimination which violates domestic laws [66].^3^

These developments bring up important questions about what is private and what is public when it comes to data, algorithms, and users—questions that must come to the fore in public debates over how governments and publics should hold technology accountable to domestic and international law, and how governments can protect publics from the potential social, economic, and health harms of technologically-driven inequities. Health researchers attempting to investigate technological determinants of health face a unique set of constraints: without understanding how algorithms are leveraged by platforms, conducting systematic, large-scale research on platforms is all but impossible, because obtaining a reliable and unbiased sample depends on understanding how algorithms inform search results. Without a reliable and robust dataset, researchers cannot create the kinds of tests that best enable them to investigate how algorithms produce disparate or inequitable outcomes [67]. Protecting public access to both de-identified data and platform algorithms is the only way that governments can ensure that technological determinants of health—and broader social effects related to technology—will be uncovered, understood, and measured.

Data transparency does not necessarily protect against privatization or potential harms, however. The self-described “non-profit startup” crowdfunding website Watsi advertises a policy of “radical transparency” in its work, even posting records of every single patient it has funded on a publicly-accessible Google spreadsheet [68]. However, these demonstrations of transparency divert attention from questions about what else may be concealed, as interviews with Watsi’s in-country medical partners have revealed: Which patients are *not* selected to be featured on the site? When patients consent to being on the site, do they know their data will be shared in this way, and what the potential impacts might be? How might stories shared on the site, or the outcomes of medical procedures, be altered in order to attract and retain donors on whom the crowdfunding site relies for survival? Watsi announced in 2018 that it had used the expertise it had developed through crowdfunding to launch a new project—Meso—which it described as “a modern technology platform for health insurance administration” [69]. This platform is now being marketed to developing countries attempting to expand their UHC. It joins the ranks of various technologies and mobile health (mHealth) platforms being marketed as health equity tools in the Global North and South [70–72]. In examining the impacts of such new technologies, we must assess not just their direct impacts on health, but also the ethics of data, privacy, and expertise upon which such tools are built.

Concerns about the crowdfunding industry’s corporatization of medical needs, and about its data ownership, marketing and transparency practices, underscore its potential impacts on the present and future health of crowdfunding customers. However, crowdfunding can also be understood as a commercial determinant, as it shapes broader social discourses and norms, determining health politics and contributing to political determinants of health for entire populations. These impacts are explored below.

### Crowdfunding as a determinant of health politics

Crowdfunding has been hailed as a “democratizing” force in fields ranging from finance and philanthropy to law and justice for the ways it increases consumer voices [73–75].^4^ However, as the preceding sections have made clear, we must assess the broader relationships of crowdfunding to political determinants of health, not just its capacity to expand consumer/donor choices. This includes, as discussed above, recognizing how political choices to expand or limit citizens’ access to health care may fuel the crowdfunding industry, and how corporate entities within that industry may continue to have an interest in limiting the expansion of UHC in order to continue profiting from medical crowdfunding campaigns.

On a larger scale, crowdfunding reflects powerful neoliberal ideologies that privilege the values of marketization, individual choice, limited government oversight, austerity in public sector programs, and the replacement of citizen entitlements with varying scales of hierarchical deservingness based on merit, identity, or market appeal [53, 76]. It would be difficult to identify an industry that more fundamentally embraces and reflects these values than crowdfunding. All platforms share a few simple premises: the privileging of individual choice, the idea that creating competitive “marketplaces” will ensure that the best causes are funded, and the importance of self-marketing and social media spread as ways for campaigns to achieve success. Even in an era where corporate norms have been overtaking many aspects of global health and public life, the rate at which the neoliberal ideals of crowdfunding have become normalized is striking.

Crowdfunding also monetizes older practices of mutual aid throughout countries in the Global South and North. Along with older technologies such as WhatsApp and M-Pesa, crowdfunding has expanded and altered traditions of mutual aid by enabling users to connect easily to broad social networks, transfer money quickly within networks, and to substitute other forms of care with monetary donations [77, 78]. Mutual aid is hardly an alternative to austerity: it often emerges as a coping strategy from within precarity. Nor does it assuage structural vulnerability: it is often insufficient for dealing with complex needs and silences contestations of the political choices that fuel such vulnerability. In the absence of more robust state support, it can create collective vulnerabilities that compound each other, as mutually dependent communities struggle to meet ballooning needs despite significant solidarity [79, 80]. Moreover, mutual aid may undermine rights- and state-based provisions, as in the case of religious health care networks in the USA, which directly challenge the Affordable Care Act [81]. Finally, for the many who arrive at crowdfunding because of the structural violence of neoliberalism but do not find success in their campaigns, crowdfunding becomes a secondary market of abandonment that conceals the baser elements of capitalist markets while reinforcing their harms.

A thorough conceptualization of the political determinants of health must also examine how factors like technology can shift the politics of health by altering discussions of rights, entitlements, and claims for recognition and redistribution are made. With crowdfunding, this means thinking about how it shifts broader public values and discourses regarding health entitlements and citizen deservingness, consequently impacting efforts to ensure the right to health. Crowdfunding has a remarkable reach in public and private discourses because of its ability to maximize traditional and new media formats to spread campaigns. It has also become a means through which citizens express allegiance with, or support for, particular identities, political causes, or activist movements [82]. Crowdfunding campaigns have become increasingly visible platforms for political discourse, as is evident in the cases of cholera funding in Zimbabwe and border-wall funding in the USA. However, the more crowdfunding is used as a platform for political discussions regarding health, the more power it gains to subtly shift public values regarding health care, entitlements, and the social contract.

Social media and digital economies in general are “disrupting the very grammar of justice,” as well as the conditions and norms upon which discussions of justice can be conducted, constituting a climate of what Nancy Fraser terms “abnormal justice” [83, 84]. At a time when political discourses seem particularly fractured and “abnormal,” crowdfunding appears to provide a platform that can reify and coalesce the norms and paradigms of public discourse. The norms that it coalesces, however, supplant the collective vision of health justice secured through a social contract with an individualized marketplace of deservingness. And the more citizens are exposed to this marketplace, or rely on it, the more do social norms appear to embrace its logics.

To illustrate this, consider the case of Luis Lang, an uninsured South Carolina man with diabetes who started a crowdfunding campaign in 2015 to cover the costs of urgently needed eye surgery due to complications from his diabetes [85]. Luis’ story was picked up by the local newspapers and then quickly went viral—though not for the reasons he had hoped. It was revealed that he was a Republican who had opposed “Obamacare,” and had not enrolled in the insurance plans offered under the new legislation [86]. That he managed to raise nearly all of his original $30,000 goal to cover his surgery was due largely to donations by supporters of the Affordable Care Act (ACA), who used his campaign page to argue that he should have signed up for insurance when he had the opportunity [87]. Many comments were quite caustic, such as this one:

Let’s re-cap shall we? You have diabetes, yet you continue to smoke. You let two open enrollment periods for the ACA go by without signing up for insurance. You were gainfully employed, yet chose to not purchase health insurance for yourself or your family. Now that you have reaped what you have sown, you expect other people to GIVE you the thousands of dollars you need for your medical procedures, preferably while you keep your $300 k house. And on top of that, you want to blame Obama for your troubles. How’s that “personal responsibility” thing working out for you? [85]

As Luis himself noted, he had fallen victim to the uneven US health-coverage landscape. Because his home state of South Carolina had refused the expansion of Medicaid under the Affordable Care Act, he fell into the all-too-common category of citizens in these states who earn too much to qualify for Medicaid but too little to get federal subsidies to help them buy private policies. His wife told reporters that they called Obamacare the “Not Fair Healthcare Act”—and in some ways they were correct, although South Carolina’s lawmakers shared responsibility for refusing the Medicaid expansion which would have alleviated this Catch-22 situation [87].

Journalists and policy experts debated the many forms of state and non-profit support which Luis was and was not qualified to receive. What passed largely unnoticed were the ways in which his story reflected key health disparities and was subjected to repeated appraisals of his deservingness. Commenters made judgements about his smoking habit and his poorly controlled diabetes; his financial assets and choices; his political views and his failure to enroll in healthcare. They criticized his appearance and his spelling, even though he apologized about them “because I can not [sic] see very well” [85]. GoFundMe provided a platform where Luis could raise money from the crowd, but also face its derision. He was helped financially, but deemed morally unworthy. In the process, a particular politics of health was normalized: one in which those with financial resources are empowered as a “crowd” to decide who should and should not deserve healthcare, and to make judgements based on assessments of behavior, ability, identity, political affiliation, and perceived worth. These political values are antithetical to the values of a truly universal health care system, where even those who do not want assistance or oppose the right to health will find themselves caught by the social safety net, should they end up plummeting towards health crisis. Few of the avowed supporters of Obamacare who commented on Luis’ case recognized that they were extolling the very same values of individual choice, free markets, and selective deservingness that have been used for decades to oppose health care reform and expansion efforts in the USA.

Ideologies of meritocracy long predate crowdfunding, but crowdfunding gives them new platforms for spread and normalization, empowering donors to become moral arbiters of who will and will not get crucial medical assistance. This may serve to invite further dismantlement of already-incomplete systems of rights-based health care and social support. Thus, crowdfunding can influence the political determinants of health, while itself becoming a technological determinant of health politics. For example, in interviews, crowdfunders express an overwhelmingly common explanation for why specific campaigns succeed, and why people should donate to their campaigns. Nearly every crowdfunder interviewed has said that campaigns succeed because the subject of the campaign is an especially “good person.” While assessments of what makes a person “good” may vary, what is reinforced across crowdfunding landscapes is a remarkable false meritocracy: people who are good deserve to be crowdfunded, and those who are successful at crowdfunding are good and deserving people. These narratives erase and obscure the innumerable inequities that shape crowdfunding access and success, and the ways in which crowdfunding technologies create inequitable outcomes. All the same, crowdfunding users readily equate crowdfunding success with goodness and deservingness, partly because of what crowdfunding normalizes and institutionalizes. In this way, crowdfunding represents not a ‘democratization’ of charity, but governing by a (prejudicial, algorithmically biased) crowd. As technology platforms co-opt public services and Mark Zuckerberg declares, “Facebook is more like a government than a traditional company,” scholars must recognize that as technology shifts cultures, economies, and norms, it is also shifting politics and taking on governance roles and institutions [88].

## Conclusion

Let us finish by returning to Zimbabwe, by way of Broad Street. In the London cholera epidemic of 1854, John Snow demonstrated the power of epidemiological deduction to challenge the prevailing idea that miasmas caused diseases like cholera, rather than pathogens. As Steven Johnson writes of the ultimate decision to remove the Broad Street pump handle,

… the pump handle … marks a turning point in the battle between urban man and *Vibrio cholerae*, because for the first time a public institution had made an informed intervention into a cholera outbreak based on a scientifically sound theory of the disease. The decision to remove the handle was not based on meteorological charts or social prejudice or watered-down medieval humorology; it was based on a methodical survey of the actual social patterns of the epidemic …. It was based on information that the city’s own organization had made visible ([89], p. 163).

Viewing these words alongside the Zimbabwean cholera campaign, we can see how crowdfunding is a deeply regressive public health strategy, abandoning sound appraisals of “actual social patterns” of need for “social prejudice.” It also removes data and decisions from the public sphere, enveloping us in a marketplace of individualized risks and choices within the broader miasmas of the global social media environment.^5^ Though crowdfunding has been greeted with significant optimism in the global health and development fields [90], this analysis shows that there are significant areas of concern about its impacts on health equity and the pursuit of UHC. The potential for harms and widening disparities is significant. This article has explored some of the most important domains in which crowdfunding may be contributing to health disparities through its role as a commercial, technological, and political determinant of health.

Because of data limitations, documenting these effects is difficult. Improved public access to data is necessary in order to document these effects. Governments can ensure better data access by curtailing the legal power enshrined in terms-of-service documents, mandating that the public has access to information on algorithms that shape access to crucial public goods (including access to information, such as on Google search), and creating data-sharing partnerships between corporations, public agencies, and research institutions.

There may be wide variation in the health and social effects of crowdfunding across platforms, across country settings, and among various communities of users. Platforms and their users vary considerably, as does engagement with those platforms across countries and regions. These possibilities underscore the need for more specific and contextualized studies of crowdfunding use and impacts. However, since many peer-based crowdfunding platforms have consistent features and similar functionality, and companies are now selling similar crowdfunding platforms to be adapted to specific markets, there likely is more similarity among platforms than might be expected as crowdfunding marketplaces expand. Certainly, these marketplaces are unevenly regulated, and subject to varying local laws in a number of different domains that can affect how the industry operates in any specific country [10]. It is likely that companies will seek to create more consistently open and unregulated markets for the expansion of crowdfunding [18]. Further independent research is urgently needed on effective regulation policies. Without better studies of how crowdfunding markets operate within specific countries (and better access to the data upon which such research depends), it will be difficult for countries to engage in this regulatory work.

Crowdfunding has the potential to provide some health benefits—a point that should not be overlooked. Notably, it enables far-flung communities (including migrant populations) to connect and provide care to those who are sick. Many interviewees report that the positive emotional benefits of crowdfunding are significant for families and patients alike, even when financial goals are not met. By amplifying the extraordinary goodwill, charitable intentions, and commitment to care of millions of people, crowdfunding underscores our collective human capacity for solidarity in support of health. However, it undermines efforts to secure health for all by refracting and fueling many of the inadequacies of existing health and social systems, and foreshadows the potentially outsized role that technology and marketization may play in future health disparities. Rather than viewing crowdfunding as a social media fad or a purely benevolent technology, researchers and publics need to recognize it as a complex innovation that is reshaping health systems, exacerbating health disparities, and shifting political norms, even as it introduces new ways of connecting and caring for those in the midst of health crises.

## Supplementary information

**Additional file 1:** Appendix A. Global Health/Medical Crowdfunding Platforms. The file provides an overview of popular global health and medical crowdfunding platforms, including the types of crowdfunding platforms used, countries served, the status of platforms as for-profit or non-profit ventures, known ties to industry, and each platform’s status in the marketplace as of January 2019.

## Data Availability

Most data generated or analyzed during this study have been included in this published article, its cited sources, and its supplementary information files. Data from ethnographic interviews and participant and online observation are not publicly available because they include sensitive and protected individual information.

## Abbreviations

ACA: Affordable Care Act
CSR: Corporate social responsibility
UHC: Universal health coverage

## Footnotes

1 GoFundMe decided in January 2019 to return all money contributed to the campaign back to donors, because of conditions that Kolfage had set for the campaign which were not met (Holcombe, 2019).

2 Not to be confused with ‘peer-to-peer’ or P2P crowdfunding, which connects peer lenders to projects. This term is used to denote and highlight the largely individual causes featured on these sites, rather than campaigns for donations to larger charitable and non-profit causes.

3 I am especially thankful to Sanya Reid Smith for directing me to this issue.

4 Of course, those who do not have the financial ability to donate to causes have no voice in these newly “democratized” public spaces.

5 I am deeply grateful to an anonymous reviewer for pointing out the connections between the Zimbabwe campaign and the Broad Street Pump history.
