# Supplementary material for: Crowdfunding and global health disparities: an exploratory conceptual and empirical analysis
Source: Global Health. 2019 Nov 28;15(Suppl 1):71. doi: 10.1186/s12992-019-0519-1 (PMC6882318; doi:10.1186/s12992-019-0519-1)
Supplement: Supplementary file 1 — Additional file 1: Appendix A. Global Health/Medical Crowdfunding Platforms. The file provides an overview of popular global health and medical crowdfunding platforms, including the types of crowdfunding platforms used, countries served, the status of platforms as for-profit or non-profit ventures, known ties to industry, and each platform’s status in the marketplace as of January 2019. [file 12992_2019_519_MOESM1_ESM.pdf]

Appendix A.

GLOBAL HEALTH / MEDICAL CROWDFUNDING PLATFORMS						
**Platform**	**Platform type**	**Fundraising categories**	**Countries served**	**For-profit status**	**Other industry ties**	**Acquisitions, mergers, closures, product launches (as of Jan 2019)**
**Backabuddy**	Peer-based and philanthropic, donation-based	Individual causes and non-profit charitable projects	South Africa	Non-profit	Provides a platform for CSR projects	
**Caringcrowd**	Philanthropic	Global public health projects run by non-profits	Projects in 44 countries	Owned and run by Janssen Global Services, LLC, parent company of Johnson & Johnson	"Sponsored" by Johnson & Johnson, used as platform for CSR activities	
**Crowdrise**	Philanthropic, donation-based	Social fundraising for non-profits	Can only fundraise for US / Canadian nonprofits; donations accepted worldwide	Private, for profit owned by Gofundme	Extensive corporate partnerships – serves as platform for CSR activities.	Acquired by Gofundme in 2017.
**Donate-Ng**	Peer-based, donation-based	Individual causes and charitable projects	Nigeria	For profit	Several corporate partnerships	
**Facebook**	Peer-based and philanthropic, donation-based	Fundraising for individual or non-profit 'causes' via the Facebook platform.	Personal campaigns can be set up in 17 European / North American countries.	For profit	Extensive platform integration for advertisers and marketing of user data.	Acquired Karma in 2012 and WhatsApp in 2014.
**Fundrazr**	Peer-based and philanthropic, donation-based	Fundraising for individual, non-profit, corporate, or social projects	Operates in all countries where PayPal, Stripe, or WePay is available.	Private, for profit, owned by Connectionpoint Systems, Inc.	Popular platform for CSR projects. Corporate partnerships with payment processors and social media companies.	
**Giveforward**	Peer-based, donation-based	Various individual causes, primarily medical	Fundraisers must be in the US or Puerto Rico	Private, for profit	Users can donate to campaigns by purchasing items of need from integrated retailers	Acquired by YouCaring in 2017 and Gofundme in 2018
**GlobalGiving**	Philanthropic, donation-based	Social and corporate	Projects in 170 countries;	Non-profit	Extensive corporate	

		fundraising for non-profits worldwide	donors in US & UK		partnerships; customizable tools and services for corporate giving and CSR	
**GoFundAfrica**	Peer-based, donation-based	Various individual causes and charities	Worldwide, based in Uganda	For profit		
**Gofundme**	Peer-based, donation-based	Various – individual and social causes; largest category is medical	Available to fundraisers in 19 countries in Europe / North America	Private, for profit company	Extensive platform integration with other social media, especially Facebook and Twitter	Acquired YouCaring, Giveforward and Generosity.com in 2018; acquired CrowdRise in 2017.
**JustGiving**	Peer-based and philanthropic, donation-based	Fundraising for individual, non-profit, corporate, or social projects	Available to registered charities in UK, US, Ireland, Australia or Canada; individual fundraisers must be UK residents	Private, for profit	Popular platform for CSR and corporate projects, offering specialized "digital solutions" for corporate initiatives	Acquired by Blackbaud in 2017
**Leetchi**	Peer-based, donation-based	Various individual causes; gifts	Worldwide – 150+ countries, based in France	For profit	Allows companies to set up 'partner' storefronts where people who have raised money can spend it. Also developed a web-based money transfer program	Top crowdfunding site in Europe; 12 million worldwide users; £1.1 billion raised by 2017
**Kangu**	Philanthropic, donation-based	Fundraising for safe births provided by non-profit medical partners in low-income countries	Medical partners in 10 low-income countries provide care	Non-profit		
**Ketto**	Various, donation- and investment-based	Individual causes, projects, and ventures; charitable projects	India-based, donations accepted worldwide	For-profit	Markets CSR and social branding for corporations as key services; corporate partnerships.	
**M-Changa**	Peer-based, donation-based	Various individual causes, medical	Kenya, expanding in east Africa	Private, for profit	Extensive integration with social media and	

		are most common			mobile money platforms	
**Milaap**	Various, donation- and investment-based	Individual causes (medical most common), charitable projects, lending	India-based, donations accepted worldwide	Private, for profit		
**Samahope**	Philanthropic, donation-based	Healthcare procedures and projects for women and children. Funding goes to providers.	11 developing countries served	Non-profit	Numerous corporate partners	Merged with Caringcrowd in 2015
**YouCaring**	Peer-based, donation-based	Various – mostly individual causes; largest category was medical	Worldwide	Private, for profit company	Extensive platform integration with other social media, especially Facebook and Twitter	Acquired by Gofundme in 2018. Prior to that, acquired Generosity.com and GiveForward.
**Watsi**	Philanthropic, donation-based	Healthcare procedures for individuals in low-income countries, via non-profit medical partners	Has supported patients in 25 low-income countries. Donations accepted worldwide	Non-profit	Funding from large tech industry firms like Y-Combinator and Tencent. Extensive corporate partnerships and opportunities for CSR activities.	Launched Meso, “a modern technology platform for health insurance administration” in 2018
